# Measurement invariance in gender and age of the Herth Hope Index to the general spanish population across the lifespan

**DOI:** 10.1007/s12144-022-03608-8

**Published:** 2022-09-08

**Authors:** María Auxiliadora Robles-Bello, David Sánchez-Teruel

**Affiliations:** 1 Department, University of Jaen, Jaen, Spain; 2 Department of Personality, Assessment and Psychological Treatment, University of Granada, Granada, Spain

**Keywords:** Hope, Herth Hope Index, Measurement invariance, Psychometric properties, Spanish population

## Abstract

The Herth Hope Index (HHI) is used to measure hope. Assessing the psychometric properties of HHI in Spanish population, exploring its structural validity, the different functionalities of the items and the invariability of this measure according to the gender and age of the population. Confirmatory factor analysis was conducted to explore the scale’s dimensionality and test for strong measurement invariance across sex and age in a cross-sectional, multicenter, prospective study. A new scale was obtained with the structure of one factor with 9 items. Goodness-of-fit indices were excellent. The internal consistency of the one dimension proved high values. The configural invariance on gender shows that both men and women understand the new HHI items, also, this research also shows that there is no scalar invariance across age groups, revealing good levels of adjustment of the item. The Spanish version of the HHI proved to be a valid, reliable instrument to assess the hope in Spanish population.

Hope is a basic psychological aspect of human well-being (Duncan et al., 2020; Pleeging et al., 2019) distinguish between cognitive and emotional hope. Larsen et al. (2020) highlighted the cognitive conception of hope, in which the person is able to show the beliefs about the ability to achieve their goals. Emotional hope is presented in Hertz’s perspective (1992), his concept is linked to general feelings of hopelessness or helplessness and is more focused on the control of emotions than thoughts. According to this author, emotional hope is a feeling that promotes positive actions, even if the adverse situation is unchangeable. Chen and Chen (2008) related Hertz’s hope scale to students’ emotional resilience. Individuals’ high hope and optimism are quick to rebound in the face of obstacles (Segerstrom, 2006; Sánchez-Teruel et al., 2020). It has been found that people who respond with positive emotions in pursuit of goals have more hope and optimism and respond with less negative effects when they encounter difficulties (Gallagher et al., 2020; Bredal & Ekeberg, 2016). In addition, greater hope was associated with greater well-being and perceived emotional control, as well as lower levels of anxiety and perceived stress by COVID-19 (Gallagher et al., 2021). An eleven-country study found high levels of hope were associated with reduced anxiety and depression; higher levels of resilient coping were associated with reduced anxiety but not depression (Ding et al., 2021).

Gasper et al. (2020) and Bryant and Cvengros (2004) suggested that hope and optimism can be considered as two indicators of a single dimension related to future orientation. Thus, these two constructions are similar but basically different, since hope is focused on the path with the motivation to achieve what is desired, and optimism concerning the general outcome expectancy of positive future results and attitude to view and interpret situations and events positively (Ginevra et al., 2017). The two components of hope are not independent, as shown by Larsen et al. (2020). Positive emotions (hope) take precedence when the person establishes predictions to face the difficulties that are present, while negative emotions (hopelessness) take precedence when the predictions suggest the impossibility of facing the situation. There are not many scales that evaluate hope including interconnection with oneself and others.

In Spain, the effects of the pandemic and the state of alarm on the mental health of the general population have been observed. In a longitudinal study (González-Sanguino et al. 2021), symptoms of depression, anxiety and post-traumatic stress disorder were assessed, along with sociodemographic data, loneliness, psychological well-being, social support and discrimination. The results showed that symptoms of depression increased significantly during confinement, decreased at the last assessment, but did not reach previous levels. With regard to anxiety there were no significant changes in the three assessments, but a downward trend was observed over time. With regard to post-traumatic stress disorder symptomatology, a downward trend was observed across the three assessments, with significantly lower scores between the first and third assessments. The importance of perceived loneliness and well-being as main predictors of mental health was also determined, as well as the importance of younger age for depression and female gender for presenting anxiety symptoms (González-Sanguino et al., 2021). In this sense, data show that the percentage of people with feelings of uncertainty, worry about suffering or contracting a serious illness or losing loved ones has increased in the Spanish population (Balluerka, 2020). Furthermore, the pandemic and confinement have led to a more negative view of the future and increased feelings of hopelessness and loneliness among the Spanish population, especially among people with symptoms of COVID-19 or a diagnosis of COVID-19, as well as those living alone, women and younger people, with a lower socio-economic status or a more precarious employment situation. Respondents also reported feeling more irritable and experiencing more anger and mood swings than before the pandemic, the latter symptom being more prevalent in women and in younger age groups. Alternatively, feelings of optimism and confidence have been reduced (especially in the female group, in people who have experienced a worsening of their employment situation or in those who have experienced symptoms or diagnosis of COVID-19), as well as feelings of vitality and energy. In relation to physical health, the study sample reported a higher prevalence of physical problems or an aggravation of existing symptoms, especially among women, people living alone and younger people (Balluerka, 2020). While the results are in line with findings from other countries such as China, Italy, Iran, the United States, Turkey, Nepal and Denmark, along with Spain, all of which had relatively high rates of symptoms of anxiety, depression, post-traumatic stress disorder, psychological distress and stress in the general population during the COVID-19 pandemic (Xiong et al., 2020), higher hopelessness was associated with higher levels of anxiety, depression, post-traumatic stress disorder, psychological distress and stress in the general population during the COVID-19 pandemic (Xiong et al., 2020). In addition, greater hope was associated with greater well-being and perceived emotional control, as well as lower levels of anxiety and perceived stress by COVID-19.

The Herth Hope Index (HHI) from Herth (1992) is composed of 12 items, with a 4 point Likert scale where 1 means completely disagree and 4 means completely agree. Items 3 and 6 are formulated in reverse, which means that their scores have to be reversed. The original factorial structure of the HHI is 3 sub-dimensions that are temporality and future, positive disposition and expectation, and interconnectedness. The maximum possible score is 48 and the minimum is 12. This scale has been translated and adapted to general Norwegian population (Rustøen et al., 2018), Japanese version (Hirano et al., 2007), Portuguese-speaking population with patients with chronic ilness (Sartore et al., 2010), Dutch version in patients with mental illness (van Gestel-Timmermans et al., 2010), Chinese version (Chan et al., 2012), Iranian elderly people (Yaghoobzadeh et al., 2019) and young Spanish people who made a suicide attempt (Sánchez-Teruel et al., 2020). All these versions have a different structure comparing to the original version. The adaptation to people who have made a suicide attempt has a two-dimensional structure (Sánchez-Teruel et al., 2020). Other adaptations result in a single factor, namely the Swedish version (Benzein & Berg, 2003), the German population with cancer (Geiser et al., 2015), also within a sample of Iranian patients with heart disease (Soleimani et al., 2019), Norwegian adults with cancer problems (Rustøen et al., 2018) and Italian patients with cancer (Ripamonti et al., 2012). This indicates that there seem to be difficulties in the structure according to the population and adaptation sample. Moreover, there are no studies evaluating the psychometric properties of this scale in the general Spanish population, nor has its invariance been evaluated according to gender or age in this population. The invariance of the measure guarantees that the evaluation instruments really measure the same construct, regardless of the characteristics of the persons or groups evaluated (Cheung & Rensvold, 2002).

The aim of this study is to evaluate the psychometric properties of HHI in the general Spanish population, exploring its structural validity, as well as the differential functioning of the items and the invariability of this measure according to the gender and the age of the population. Furthermore, its inverse relationship with psychopathological states like anxiety and depression and its positive relationship with protective factors such as dispositional optimism will be assessed.

## Method

### Participants

The total sample was 1,544 persons where 783(50.71%) were female and 761(49.29%) were male with ages ranging from 15 to 73 years (M = 31.12; SD = 9.23). The inclusion criteria were: (1) to be 15 years old or over (2) to have Spanish nationality and be legally resident in Spain (3) to have read the information sheet and accepted the informed consent document and (4) to have completed all the questionnaire. Table 1 presents the socio-demographic data of the sample. The sample was heterogeneous and representative of the Spanish population (Centre for Sociological Studies-CIS., 2020). No significant statistical differences were found by sex or age group.

**Table 1:** Description of socio-demographic data of the sample

	N(%)	Contrast	η^2^
Gender			
Women	783(50.71)	2.84^ns^	0.79
Men	761(49.29)		
Age			
15–17	214(13.86)		
18–38	412(26.68)		
39–59	484(31.35)	1.43^ns^	0.85
60–73	434(28.11)		
Number of inhabitants place of residence			
<5.000	271(17.55)		
5.000-24.999	358(23.19)		
25.000-49.999	364(23.57)	2.21^ns^	0.71
50.000-100.000	285(18.46)		
> 100.000	266(17.23)		
Level of education completed			
None	461(29.86)		
Secondary education	370(23.96)	4.12**	0.54
Bachelors degree / Vocational training	469(30.38)		
Post-graduate qualification	244(15.80)		
Employment situation			
Employed	425(27.52)		
Self-employed	356(23.06)	2.81^ns^	0.73
Retired	381(24.68)		
Unemployed/FTRE/Student	382(24.74)		
Total	1,544(100)		

FTRE = Files for Temporary Regulation of Employment; Contrast = *T*-Student/Chi-Square; * = *p* < .05; ** = *p* < .01; ns = Not significant; d.f. = degree of freedom; η2 = eta square

### Instruments

*Socio-demographic data sheet*. We prepared a fact sheet for this study to capture information on sex, age, location, educational level and employment status.

*Herth Hope Index-HHI by* Herth (1992) based on the hope model of Dufault and Martocchio (1985). The Spanish translated version was used for a multi-professional sample (Meseguer et al., 2013). This scale measures hope using 12 Likert-type items (1 = completely disagree; 4 = completely agree), covering three factors of four items each as in the original English version: (a) temporality and future as a cognitive-temporal dimension of hope that assesses thoughts related to the likelihood of a desired future outcome (sum of items 1, 2, 6 and 11); (b) positive readiness and expectancy as an affective-behavioural dimension it measures the confidence necessary for the initiation of action (sum of items 4,7,10 and 12); and (c) interconnectedness as an affiliative-contextual dimension it measures the relationship (positive or negative) between the person and him/herself and others (sum of items 3,5,8 and 9). Items 3 and 6 are scored inversely. The original study found the scale to have adequate psychometric properties (alpha = 0.97; test-retest = 0.91) and a three-dimensional structure following the hope model (Dufault & Martocchio, 1985), although recent studies in the Spanish clinical population (people with previous suicide attempts) show a two-dimensional structure and a Cronbach’s alpha of 0.97 (Sánchez-Teruel et al., 2020).

*Life Orientation Test-LOT-R* by Scheier et al. (1994). We used the Spanish adaptation by Ferrando et al. (2002). The instrument is made up of 10 items, with a 5-point response scale where 0 is completely disagree, and 4 is completely agree. Of the 10 items, only 6 measure aspects related to dispositional optimism, while the others are fillers. Of these 6, three are written positively and three negtively, such that it produces one score related to optimism or life orientation and another score that measures pessimism. Cronbach’s alpha for the adaptation to Spanish was 0.70 for optimism and 0.69 for pessimism.

*Hospital, Anxiety and Depression (HAD-14)* by Zigmond and Snaith (1983) in its Spanish version by Herrero et al. (2003). A 14-items scale was designed for the assessment of anxiety and depression in non-psychiatric outpatient hospital services. It is a state measure containing two scales, one for anxiety and another for depression. One of its main strengths is the suppression of somatic symptoms so that it can be assessed independently of the underlying somatic disease. It is a useful instrument validated in our environment, and of special interest and relevance in the context of Primary Care. It presents a subscale of anxiety of 7 items and a subscale of depression of 7 items in a 4 point Likert type format giving the maximum subscale scores of 21 for both depression and anxiety subscales. The questionnaire evaluates the symptoms during the previous week. This scale has a good internal consistency of 0.90 according to Cronbach’s alpha for the full scale; 0.84 for the depression subscale and 0.85 for the anxiety subscale (Herrero et al., 2003). In this study, the alpha on the value of the total inventory was 0.86 and they were also adequate for the remaining sub-dimensions (α_Anxiety_ = 0.89; α_Depression_ = 0.83).

### Procedure

Firstly, the research ethics committee of the University of Jaen (Spain) was asked to approve the study. Secondly, permission was sought to use the original author scale for the adaptation of the HHI. Subsequently, was translated the original version of the HHI to Spanish through a standardized translation process (Gjersing et al., 2010). Two bilingual experts (English-Spanish) and two external translators were asked to translate the HHI into Spanish based on the rules set out by the International Test Commission (Hambleton, 2005). These translations were revised and translated back into English (Gudmundsson, 2009) by a bilingual Doctor of Psychology, unrelated to this research who made the appropriate terminological adjustments in some terms not agreed upon by the previous translators, and sent the final version of the instrument in Spanish. All the instructions given for adaptation of evaluation instruments in psychology were properly followed (Muñiz & Fonseca-Pedrero, 2019). Finally, data collection was carried out from 22 to 2022 through an online survey (Google Forms, licensed by the University of Jaen) which was disseminated through social networks and mobile media. Participants completed the informed consent and all the questionnaires in Spanish. Data was collected from participants under the age of 18 through the social networks of the friends of the authors’ parents, with children of similar ages. The information was sent to the parents and the parents sent it to their children, if that was their wish.The study was approved by the ethics committee of the University of Jaen (code: ABR.20/4.PRY), and followed the ethical guidelines of the Spanish Society of Psychology and the principles of the Declaration of Helsinki.

### Data analysis

Missing data accounted for less than 1%, and the Hot-Deck Multiple-Input method was applied (Lorenzo-Seva & Van-Ginkel, 2016). First, the descriptive analysis of the items was carried out and a confirmatory factor analysis (CFA) was then performed with the original three-dimensional version (Model 1), of the two-dimensional version in the Spanish clinical population (Model 2) and of one-dimensional version the Spanish general population subjected to compulsory confinement by COVID-19 (Model 3). In the analyses with the three-dimensional (Model 1), two-dimensional (Model 2) and one-dimensional (Model 3) structure, very low factor loadings were obtained in three items (3, 5 and 6). Therefore, it was also decided to analyse the unidimensional version in Spanish general population subjected to compulsory confinement by COVID-19 with the elimination of these items (Model 4). The measurement models with more than one factor allowed correlations between factors. For confirmatory analyses, the polychoric correlation matrix with the generalised least squares (GLS) method was used. The fit indices used the ratio χ^2^/df, the approximation mean square error (RMSEA), the comparative fit index (CFI), the Goodness of fit index (GFI), the Tucker-Lewis index (TLI) and the Expected Cross Validation Index (ECVI). The goodness-of-fit model was considered satisfactory when the TLI and the CFI ≥ were 0,95, Goodness of fit index (GFI) is close to 0.90 and the RMSEA approached 0,05 (Kline, 2016). For an acceptable model it χ2/df should be between 2 and 3 and for a good model it between 0 and 2 (Schermelleh-Engel et al., 2003). The χ2/df ratio was used rather than the chi-square test statistic because it is less sensitive to sample size (Schermelleh-Engel et al., 2003). The Expected Cross Validation Index (ECVI) indicates the discrepancy between the covariance matrix of the sample analysed and the expected matrix that would be obtained in another sample of equivalent size (Browne, 2000; Browne & Cudeck, 1993). The ECVI compares different models, considering that the model with the smallest value will exhibit the best potential for replication (De Jongh et al., 2011; Vega-Gea et al., 2016). Secondly, also analyzed whether there were differences in the invariance of the measure by gender and age using multi-group CFA with AMOS. Two nested models for gender and four models for age were defined. Specifically, the Satorra-Bentler scale (χ^2^) and its p-values, along with RMSEA with 90% CI and CFI, were used for the invariance of the measure as an incremental adjustment index (Hooper et al., 2008). There is invariance of the measure when the p > .05 of Δχ^2^ (considering the sample size bias); the RMSEA values ≤ 0.05 and the ΔCFI value of the models compared is < 0.01 (Byrne, 2016). An analysis of configural invariance (baseline model) was conducted to test whether groups associate the same subsets of items with the same constructs, with the factor means set to zero. As well as metric invariance to check whether the factor loadings between each item and its factor are the same in all groups. And scalar invariance to assess whether the differences between the groups indicated by the items are the same for all items (Cheung & Rensvold, 2002; Van de Schoot et al., 2012). Finally, data on the divergent validity of the resulting instrument was obtained by calculating Pearson’s correlation coefficients with LOT-R and the HAD-14 scale and we also evaluated reliability using the internal consistency procedure (Cronbach´s alpha and McDonald’s omega coefficients). All analyses were performed using SPSS 23 AMOS (IBM Corporation, 2013) and jMetrik (Meyer, 2014) and the level of statistical significance required in all tests was a minimum of *p* < .05.

## Results

The average scores of the HHI items were more higher than the theoretical midpoint of the scale (i.e. 2). The lowest average was at item 5 (M = 2.20) and its deviation standard was the highest (SD = 1.04), and item 7 showed the highest mean (M = 3.44; SD = 0.47). The correlation between item and total is low for item 5 (0.24), and low and negative for items 3 (-0.26) and 6 (-0.19). The reliability of internal consistency, estimated by the ordinal alpha, was 0.62 for the total sample; this value improves with the elimination of elements 3, 5 and 6 (Table 2).

**Table 2:** Descriptive statistics, skewness and kurtosis indices, and item analysis of the Herth-Herth Index of Hope (HHI) (n = 1,544)

	M(SD)	*K-S*	S	K	*r item-total*	*α if item deleted*
			*SE(0.09)*	*SE(0.17)*		
Item 1	3.03(0.86)	0.25**	− 0.61	− 0.27	0.57	0.60
Item 2	3.29(0.78)	0.28**	− 0.94	0.43	0.51	0.62
Item 3	3.20(0.92)	0.29**	− 0.88	− 0.26	− 0.26	0.70
Item 4	3.02(0.70)	0.30**	− 0.46	0.31	0.56	0.61
Item 5	2.20(1.04)	0.19**	0.20	-1.22	0.24	0.69
Item 6	2.45(0.98)	0.20**	0.05	− 0.99	− 0.19	0.77
Item 7	3.44(0.47)	0.40**	-1.58	-2.18	0.48	0.64
Item 8	2.92(0.77)	0.28**	− 0.37	− 0.20	0.54	0.61
Item 9	3.43(0.78)	0.34**	-1.35	1.36	0.51	0.61
Item 10	3.20(0.84)	0.25**	− 0.86	0.12	0.56	0.63
Item 11	3.29(0.81)	0.29**	− 0.98	0.36	0.64	0.62
Item 12	3.48(0.74)	0.36**	-1.40	1.54	0.65	0.62
Total	37.18 (6.25)	0.09**	− 0.82	0.70	1	0.62

M = Mean; SD = Standard deviation; S = Skewness; K = Kurtosis; SE = Standard error of skewness and kurtosis; K-S = Kolmogorov-Smirnov test; ^*^Significant correlation at the 0.05 level (bilateral); ^**^Significant correlation at the 0.01 level (bilateral)

### Dimensionality and factor structure (n = 1,544)

**Fig. 1:**
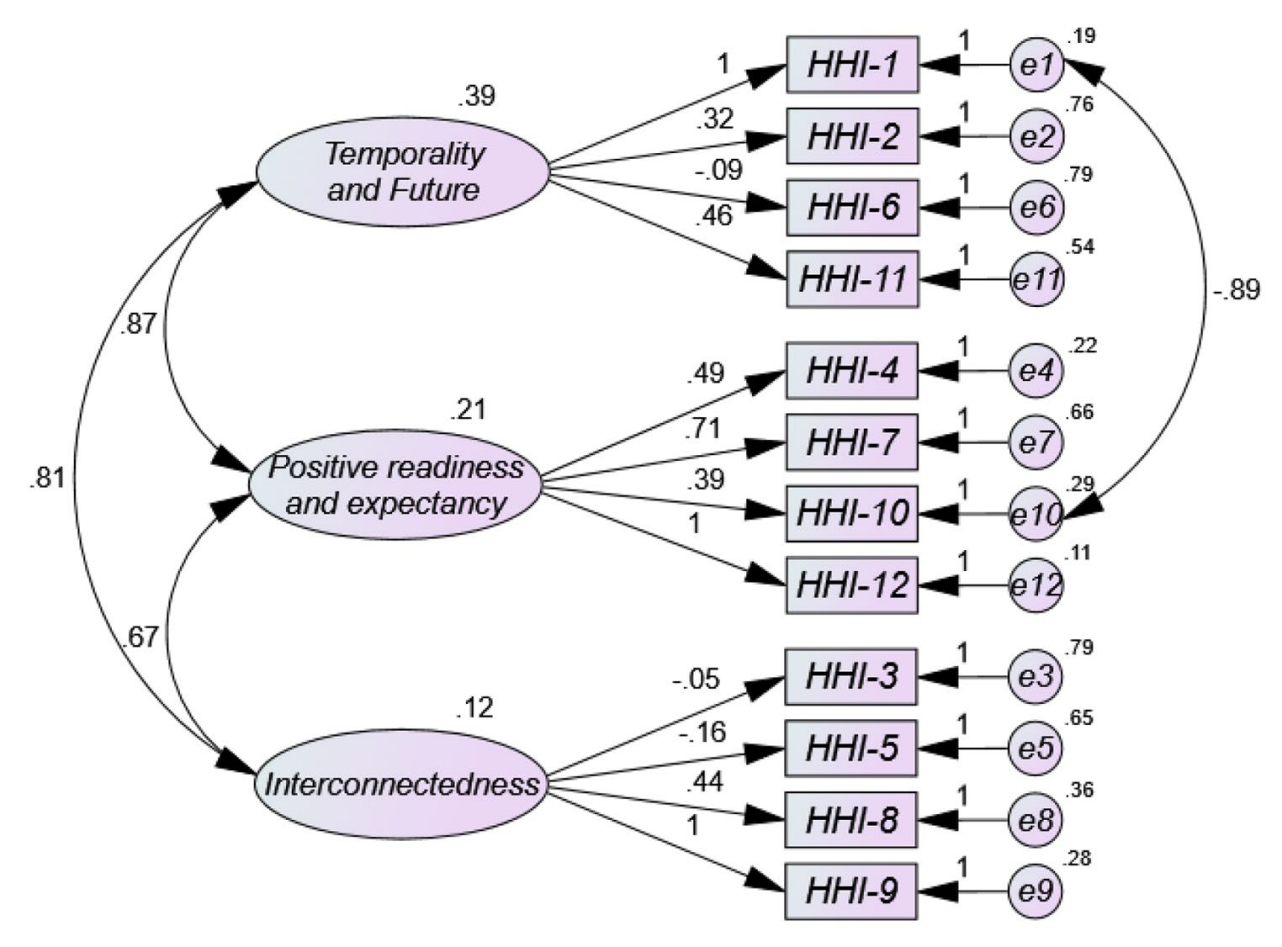
Path diagram of the original three-dimensional version (Model 1 = Meseguer et al., 2013)

**Fig. 2:**
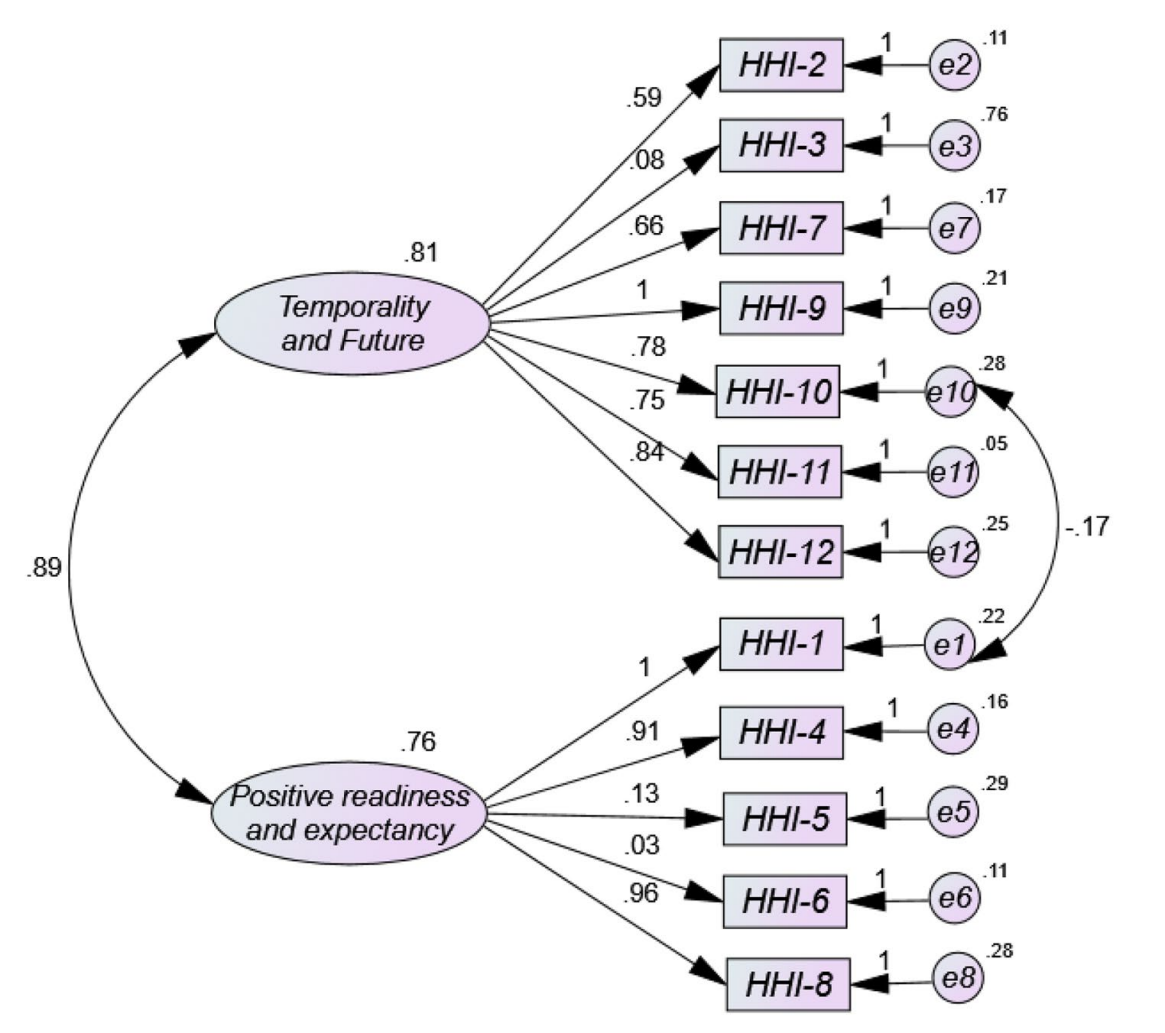
Path diagram of the two-dimensional version in the Spanish clinical population (Model 2 = Sánchez-Teruel et al., 2020)

**Fig. 3:**
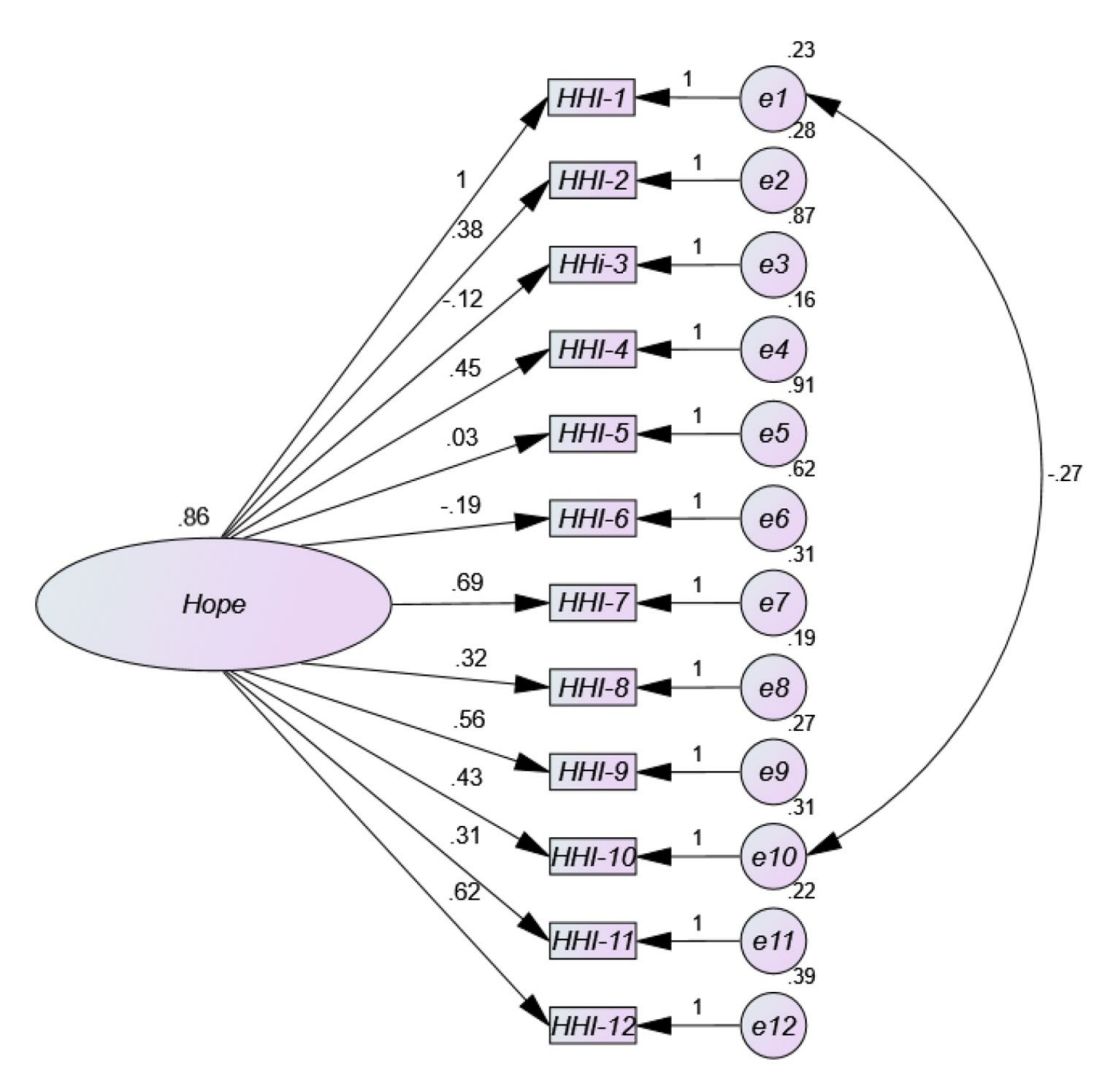
Path diagram of the one-dimensional model corresponding to the HHI in Spanish general population subjected to compulsory confinement by COVID-19 (Model 3)

**Fig. 4:**
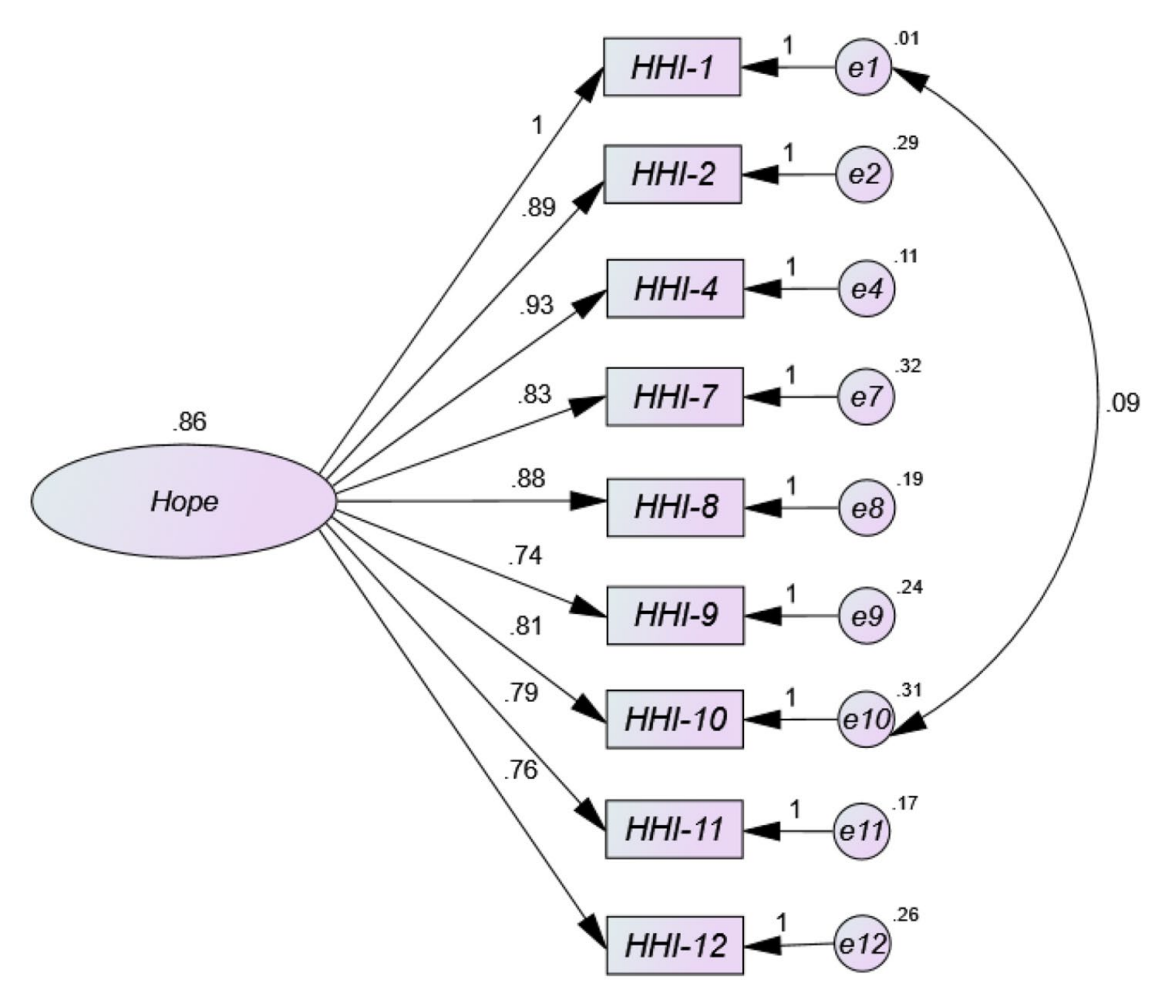
Path diagram of the one-dimensional model corresponding to the HHI in Spanish general population subjected to compulsory confinement by COVID-19 (Model 4 = eliminating items 3, 6 and 5)

The results obtained from the univariate and multivariate normality analyses showed that there was neither univariate nor multivariate normality in item distribution (Mardia = 421.17; (Mardia, 1970). Figure 1 three-factor Model 1 shows low factor loads (< 0.50) for most items in the HHI path diagram. As can be seen, the standardized weight values (coefficients from β) ranged from 0.32 for item 2 to 0.71 for item 7, with extremely low and negative loadings on item 3 (-0.05), item 5 (-0.15) and item 6 (-0.09). As for the correlation estimates between Temporality and Future (TF) and Positive Readiness and Expectancy (PRE) it is high (0.87), as well as between Temporality and Future (TF) and Interconnectedness (0.81), and adequate between Positive Disposition and Expectation (PRE) and Interconnectedness (0.67). The parameter values of the two-factor solution (Model 2) (Fig. 2) are more adequate than model 1, with the same exception of items 3, 5 and 6, with factor loadings of 0.08, 0.13 and 0.03 respectively. The remaining values range between 0.59 and 0.96. The estimated correlation between the two resulting factors Temporality and Future (TF) and Positive Readiness and Expectancy (PRE) is very high (0.89). Model 3 (Fig. 3) with unidimensional structure in its full version also shows low factor loadings between 0.31 and 0.62, but in addition, items 3, 5 and 6 offer extremely low factor loadings (-0.12; 0.03; − 0.19). However, the one-factor model 4 (Fig. 4) where items with loadings below 0.50 (items 3, 5 and 6) were removed was the most appropriate, offering values above 0.50 (between 0.76 and 0.93) of factor saturation for the remaining nine items. Furthermore, the correlations between the factors in the two- and three-dimensional models are very high, indicating the presence of a large common factor.

Looking at the comparison between the four proposed models (Table 3), there are differences between χ^2^/df as a goodness-of-fit index, with 0 and 2 indicating a very good fit (Schermelleh-Engel et al., 2003). However, there is increasing agreement on the ΔCFI as the basis for assessing whether the model fit is significantly better (Meade et al., 2008), with the nine-item single-factor model obtaining the best fit to this sample based on the increase in CFI. In addition, goodness-of-fit indices show that the reduced version of nine items produced very good indexes of goodness-of-fit of HHI in this population. Based on these results, the acceptability and goodness-of-fit of this model is considered strong. Therefore, the data confirm a one-dimensional structure with 9 items of HHI in this sample of the general Spanish population subjected to mandatory home confinement by the Government as a COVID-19 prevention measure.

**Table 3:** Comparison of the goodness-of-fit indices of the four factorial structures proposed for HHI

*Model*	χ^2^	df	χ^2^/df	*p*	RMSEA (IC95%)	CFI	TLI	GFI	ECVI
1	1534.35	47	32.65	0.00	0.06[0.05; 0.07]	0.91	0.93	0.85	3.56
2	138.41	48	2.88	0.00	0.03[0.02; 0.04]	0.97	0.96	0.90	1.91
3	1438.12	46	31.26	0.00	0.05[0.04; 0.06]	0.95	0.94	0.89	2.78
4	83.14	48	1.73	0.11	0.02[0.01; 0.03]	0.98	0.99	0.97	1.23

Model 1 = original three-dimensional version; Model 2 = Two-dimensional version in the Spanish clinical population; Model 3 = Full unidimensional version in the Spanish general population subjected to compulsory confinement by COVID-19; Model 4 = Unidimensional version eliminating items 3, 5 and 6 in the general Spanish population subjected to compulsory by COVID-19; χ^2^ = Chi-square; df = degrees of freedom, χ^2^/df = Chi-square goodness-of-fit index; p = significance level; RMSEA = Approximation mean square error; CFI = Comparative fit index; TLI = Tucker-Lewis index; GFI = Goodness of fit index; ECVI = Expected Cross Validation Index

### Measurement invariance (n = 1,544)

Taking into account the results of the CFAs for the tested measurement models, the measurement invariance (MI) analyses focused on the measurement model of the 9-item version called HHI-s (Appendix 1), which was the only one that fitted the data optimally. The results of the MI are presented in Table 4, wherein it is noted that CFA models specified for males and females and for each age group demonstrated a good fit to the data, indicating that a multiplegroup CFA was appropriate. The test of configural (baseline model = factor loadings and variances were freely estimated for men and women and for each age group), metric invariance (factor loadings were constrained to be equal across gender groups and age groups) and scalar invariances (all item intercepts are forced equal for all items) also revealed good levels of fit. In summary, strong invariance was clearly achieved between gender, but not with age. Specifically, with respect to gender the increase in χ^2^ from the base model to the total metric invariance model was 1.44 (Δχ^2^ = 1.44(Δdf = 2); p > .05), a change that is not statistically significant. Likewise, the increase in CFI is 0.001 below the 0.01 criterion (Cheung & Rensvold, 2002; Van de Schoot et al., 2012), so both indices point to total metric equivalence between men and women. Likewise, metric invariance seems to show that there is no significant variation in expectancy across the age groups presented (Δχ^2^(Δdf = 2) = 4.16; p > .05), and the incremental ΔCFI is below the criterion. The attainment of this MI level allows us to assume that the same construct is measured in the different gender and age groups and that the regression parameters of the HHI-s items are the same (i.e., invariant) in these groups. However, as can be seen in Table 4, the comparison of the base model with the scalar model in age is statistically significant (Δχ^2^(Δdf = 6) = 111.57; p < .01), the increase in CFI being 0.04, which is well above the recommended maximum, so that full scalar equivalence between each age group cannot be established.

**Table 4:** Fit indices for the invariance test in gender and age

	χ^2^	df	*p*	RMSEA (95%CI)	CFI	Δχ^2^	ΔCFI	
Men (n = 761)	41.07	26	0.01	0.03[0.010; 0.042]	0.95			
Women (n = 783)	44.22	26	0.05	0.04[0.031; 0.052]	0.96			
Configural invariance gender	107.52	44	0.27	0.02[0.012; 0.031]	0.97			
Metric invariance gender	108.96	46	0.29	0.02[0.012; 0.032]	0.97	1.44^ns^(Δdf = 2)	0.001	
Scalar invariance gender	110.23	51	0.12	0.03[0.022; 0.046]	0.96	1.27^ns^(Δdf = 5)	0.002	
Age(15–17)	137.11	61	0.01	0.01 [0.001; 0.023]	0.95			
Age(18–38)	129.14	53	0.00	0.01 [0.004; 0.019]	0.96			
Age(39–59)	135.22	58	0.00	0.02[0.019; 0.034]	0.98			
Age(60 or more)	161.18	71	0.01	0.03[0.026; 0.047]	0.95			
Configural invariance age	185.21	92	0.87	0.01[0.001; 0.026]	0.95			
Metric invariance age	189.37	94	0.32	02[0.029; 0.031]	0.95	4.16^ns^(Δdf = 2)	0.001	
Scalar invariance age	296.78	98	0.11	04[0.031; 0.056]	0.99	111.57^**^(Δdf = 6)	0.04	

*χ*^*2*^ = Chi-square; df = degrees of freedom, p = significance level; RMSEA = Root mean square error of approximation; CFI = Comparative Fit Index; Δχ^2^ = Difference test between the configural and metric or scalar invariance models; ΔCFI = Difference test between Comparative Fit Index; * = *p* < .05; ** = *p* < .01; ns = Not significant

### Divergent validity and reliability

The results shown in Table 5 show that there is a positive correlation between hope and dispositional optimism (r = .82) and high inverse correlations between hope and anxiety and between hope and depression, although to a greater extent with the latter (r_a_ = − 0.86; r_d_ = − 0.92). Finally, the HHI-s with 9 items presents a high reliability in this subsample of Spanish general population.

**Table 5:** Divergent validity with anxiety and depression, convergent validity with optimism and reliability

	HAD-14	Anxiety	Depression	LOT-R	ω	α
HHI-s	− 0.89**	− 0.86**	− 0.92**	0.82	0.86	0.90

HHI-s = Herth Hope Index adapted to general Spanish population (9 items); HAD-14 = Hospital, Anxiety and Depression; LOT-R = Life Orientation Test (Dispositional optimism); ω = McDonald’s omega coefficients; α = Cronbach´s alpha test; * = *p* < .05; ** = *p* < .01; ns = Not significant

## Discussion

The aim of this study was to evaluate the psychometric properties of HHI in the general Spanish population, exploring its structural characteristics and confirming the most appropriate structure in this subsample. In addition, the differential functioning of the item, the invariance of the measure according to gender and age, and the relationship with psychopathological states like anxiety and depression and protective factors such as dispositional optimism were assessed.

It should be noted that there is a need to better understand this type of construct within a variety of different intercultural contexts (Ripamonti et al., 2012), to ensure that the translation of a scale is not just that but culturally sensitive and psychometrically sound in order to generate valid and generalizable research results (Chan et al., 2012).

In the confirmatory analysis of the different dimensions (three-dimensional, two-dimensional and full one-dimensional) of the HHI, three items (3, 5 and 6) were found to have very low or even negative factor loadings in three of the four models analysed. The results of this study have shown with model four the unifactorial structure of the HHI without these three items, enhancing the consistency of the goodness-of-fit indices and producing a considerable increase in the reliability of this short nine-item scale in a Spanish population subjected to an adverse situation related to government-mandated confinement as a COVID-19 prevention measure. Other investigations have also shown the problems of different items of the HHI, in particular Benzein and Berg (2003), Chan et al. (2012) and Van Gestel-Timmermans et al. (2010) found problems in items 4 and 5 named it “Religiosity”. Rustøen et al. (2018) reduced the HHI to 7 items by eliminating items 3, 4, 5, 6, and 7, and Soleimani et al. (2019) had problems with item 6. Specifically, items 3 and 6 are inverse and can cause problems because the self-reported measurement method may cause measurement errors. Conversely, measurement errors can be the consequence of using similar words and expressions in both positive and negative statements (Yaghoobzadeh et al., 2019). In addition, multiple studies have not been able to replicate the three-factor model (Benzein & Berg, 2003; Geiser et al., 2015; Rustøen et al., 2018; Ripamonti et al., 2012; Sánchez-Teruel et al., 2020; Soleimani et al., 2019).

None of the known studies had assessed the age and gender invariance of the HHI, and no studies have found differences in the HHI due to these variables. The results of the measurement invariance, wherein it is noted that CFA models specified for males and females and for each age group demonstrated a good fit to the data, indicating that a multiplegroup CFA was appropriate. The metric and configural invariance on gender shows that both men and women understand the HHI-s items in the same way, revealing good levels of adjustment. Similarly, the comparison of groups according to age seems to show that there is variation in HHI according to the age brackets presented. Therefore, it can be concluded that there is invariance of the measure with respect to gender but not the age in this sample of the general Spanish population. Developmental researchers tend to think of single individuals and the way their traits or performance change across time or age, what they usually examine are the data of groups of persons. In such data, several parameters can be used to describe the distribution of performance differences and their associations across time or age in the HHI. Typical parameters are means, variances, and covariances, all of which may differ across age. The question of whether the hope changes across the lifespan or whether it remains stable can, thus, be answered with this research. These data are in line with previous meta-analysis work in which no discrepancies have been found in the variables that predict the sex in relation to hope (Yarcheski & Mahon, 2016). However, this research also shows that there is no scalar invariance across age groups. This may be explained by the fact that one age group interprets the HHI-s items differently from the others. Additionally, results show that age and better educational opportunities were associated with protection (i.e.resilience and hope) and emotional well-being (i.e. affective symptoms and hopelessness) (Morote el al., 2017). This makes us reflect on the importance of applying this scale with some caution in very young or very old people, even in a pandemic situation due to covid-19, so the strategies that mitigate COVID-19 exposure and enhance hope and resilience may reduce anxiety and depression during global emergencies (Ding et al., 2021). Age is an issue that needs to be studied further in order to provide more age-adjusted tests.

It is time to discard the focus on risk factors such as hopelessness, and focus on protective factors such as hope for the assessment of patients’ mental health. We offer a new scale adapted to the general Spanish population, short and easy to apply, for example, in hospital emergency rooms and clinical or psychosocial services that could assess their level of vulnerability to adverse situations. The total hope scores can be used to get an idea of where the patients are at the time of their visits. Having this information about a patient’s goal-oriented, hope-driven energy has the potential to make empathic connections easier and create opportunities to ask specific questions based on a patient’s strengths and capabilities related to making a health behavior change (Duncan et al., 2020).

Finally as expected, on the grounds of the results, there is a positive correlation between hope and dispositional optimism (Bryant & Cvengros, 2004; Ginevra et al., 2017) and high inverse correlations between hope and anxiety (Ciarrochi et al., 2014) and between hope and depression (Rand, 2017) the same is true when the population is adolescent (Yarcheski & Mahon, 2016). The HHI-s with 9 items presents a high reliability in this sample of Spanish general population.

### Limitations

First, the main difficulty is that the generalization of the results is compromised due to the use of the sampling method used (Simons, Shoda & Lindsay, 2017). Another limitation has to do with the type of model used, however, relevant authors such as Kottorp and Petersson (2011) consider that the choice of model has little impact on the validity of the findings. Therefore, in the future it would be interesting to carry out longitudinal studies and also in different types of populations with different disease processes or exposed to various adverse situations to observe how they deal with all these situations.

## Conclusion

It is important to find an assessment test of hope in the Spanish population that is not a university population because hope is important to face the obstacles or difficulties of daily life, as well as situations of illness. Additionally, if that population is suffering from mandatory home confinement due to a global pandemic, it may be meaningful to measure their level of hope. In such a way that if we know which are the people with high levels of hope we can know if they are more predisposed to perceive symptoms and signs of loss of their health and to act from prevention since they possess high levels of psychological well-being (Herth, 1992).

A reduced 9-item HHI scale from the present sample demonstrates better psychometric properties (unidimensionality) and a similar level of precision as the original HHI. The HHI was translated into the Spanish language and the general population was tested for reliability and validity using a convenience sample of a healthy person. To our knowledge, this is the first study that attempted to assess the validity and reliability of the scale and confirming the most appropriate structure in this sample of Spanish populatión. In addition, the invariance of the measure according to gender and age are aspects to be considered from an applied point of view. The HHI is reliable in assessing men and women from age 15 to 73 and and shows good internal consistency and a significant positive relationship with optimism and a negative relationship with anxiety and especially with depression.

## Data Availability

The datasets generated during and/or analyzed during the current study are not publicly available to assure the confidentiality and anonymity of the participants, due to the privacy of the same for further research, but are available from the corresponding author on reasonable request.

## Appendix 1

### Hert Hope Index in Spanish-HHI-S

**This scale has been designed to be completed by general Spanish population from 15 to 73 years old**

Lea cuidadosamente cada una de las siguientes afirmaciones. A continuación, elija la respuesta que mejor describa su opinión. 1 = Totalmente en desacuerdo; 2 = En desacuerdo; 3 = A veces de acuerdo; 4 = Totalmente de acuerdo / Please read each of the following statements carefully. Then choose the answer that best describes your opinion. 1 = Strongly disagree; 2 = Disagree; 3 = Sometimes agree; 4 = Strongly agree.

1. Soy optimista acerca de la vida / I am optimistic about life.
2. Tengo planes a corto y a largo plazo / I have short and long term plans.
3. Puedo ver posibilidades en medio de las dificultades / I can see possibilities in the midst of difficulties.
4. Puedo recordar los momentos felices y agradables / I can remember the happy and pleasant moments.
5. Me siento muy fuerte / I feel very strong.
6. Me siento capaz de dar y recibir afecto o amor / I feel capable of giving and receiving affection/love.
7. Sé dónde quiero ir /I know where I want to go.
8. Yo creo en el valor de cada día / I believe in the value of every day.
9. Valoro mi vida / I value my life.
